# Effects of waveform shape and electrode material on KiloHertz frequency alternating current block of mammalian peripheral nerve

**DOI:** 10.1186/s42234-022-00093-z

**Published:** 2022-07-27

**Authors:** David B. Green, Joseph A. Kilgore, Shane A. Bender, Robert J. Daniels, Douglas D. Gunzler, Tina L. Vrabec, Niloy Bhadra

**Affiliations:** 1 Department of Physical Medicine and Rehabilitation, MetroHealth Medical Center, Cleveland, OH USA; 2 Department of Physical Medicine and Rehabilitation, School of Medicine, Case Western Reserve University, Cleveland, OH USA; 3 Department of Medicine, Population Health Research Institute, Center for Healthcare Research & Policy, MetroHealth Medical Center, Cleveland, OH USA

**Keywords:** Nerve conduction block, KiloHertz frequency alternating current, High frequency, Waveform shape, Electrode material, High capacitance electrodes

## Abstract

**Objectives:**

KiloHertz frequency alternating current waveforms produce conduction block in peripheral nerves. It is not clearly known how the waveform shape affects block outcomes, and if waveform effects are frequency dependent. We determined the effects of waveform shape using two types of electrodes.

**Materials and methods:**

Acute in-vivo experiments were performed on 12 rats. Bipolar electrodes were used to electrically block motor nerve impulses in the sciatic nerve, as measured using force output from the gastrocnemius muscle. Three blocking waveforms were delivered (sinusoidal, square and triangular) at 6 frequencies (10–60 kHz). Bare platinum electrodes were compared with carbon black coated electrodes. We determined the minimum amplitude that could completely block motor nerve conduction (block threshold), and measured properties of the onset response, which is a transient period of nerve activation at the start of block. In-vivo results were compared with computational modeling conducted using the NEURON simulation environment using a nerve membrane model modified for stimulation in the kilohertz frequency range.

**Results:**

For the majority of parameters, in-vivo testing and simulations showed similar results: Block thresholds increased linearly with frequency for all three waveforms. Block thresholds were significantly different between waveforms; lowest for the square waveform and highest for triangular waveform. When converted to charge per cycle, square waveforms required the maximum charge per phase, and triangular waveforms the least. Onset parameters were affected by blocking frequency but not by waveform shape. Electrode comparisons were performed only in-vivo. Electrodes with carbon black coatings gave significantly lower block thresholds and reduced onset responses across all blocking frequencies. For 10 and 20 kHz, carbon black coating significantly reduced the charge required for nerve block.

**Conclusions:**

We conclude that both sinusoidal and square waveforms at frequencies of 20 kHz or higher would be optimal. Future investigation of carbon black or other high charge capacity electrodes may be useful in achieving block with lower BTs and onsets. These findings will be of importance for designing clinical nerve block systems.

## Introduction

Continuous charge-balanced kilohertz frequency alternating current (KHFAC) waveforms have been shown to produce a rapidly acting and quickly reversible nerve conduction block with minimum side effects (Tanner 1962; Woo and Campbell 1964; Bowman and McNeal 1986; Kilgore and Bhadra 2004; Tai et al. 2004; Bhadra and Kilgore 2005; Williamson and Andrews 2005; Patel and Butera 2015; Peña et al. 2020). These features of KHFAC nerve conduction block provide an opportunity for the amelioration or control of diseases where the pathological generation of peripheral nerve impulses is a major disabling factor, such as in spasticity, abnormal movements (choreas and tics), nerve pain of peripheral origin and some autonomic conditions. Existing pharmacological, chemical, and surgical methods for treating these conditions are slow acting, non-reversible and/or have systemic side-effects. Therefore, KHFAC nerve block has been proposed as an alternative method for treatment in many of these conditions (Kilgore and Bhadra 2004; Tai et al. 2004; Bhadra and Kilgore 2005; Ray et al. 2021; Dewberry et al. 2021; Jiman et al. 2019).

KHFAC nerve block utilizes alternating electrical currents in the frequency range of 1 kHz to 60 kHz and possibly higher, as summarized in Table 1 (Woo and Campbell 1964; Bowman and McNeal 1986; Tai et al. 2004; Bhadra and Kilgore 2005; Williamson and Andrews 2005; Patel and Butera 2015; Bhadra et al. 2006; Joseph and Butera 2011; Cuellar et al. 2013). The amplitudes (in Volts) required to produce conduction block vary, depending on the electrode design, nerve size and waveform frequency. Accurate comparison between published papers is hampered because it is often not reported whether waveform amplitudes are measured using peak (p) or peak to peak (pp) amplitudes, with the potential for a two-fold inaccuracy. The KHFAC is delivered directly to the peripheral nerve through electrodes surrounding the nerve and produces a localized region of conduction block through which action potentials cannot pass. The conduction block is maintained for as long as the KHFAC is delivered. Upon cessation of the KHFAC, the nerve usually resumes normal action potential conduction, unless the KHFAC duration produces a carry-over block effect as a result of prolonged block (Bhadra et al. 2018). Experimentally, it has been demonstrated that the block is effective within milliseconds and can be reversed within 1 s (Bhadra and Kilgore 2005; Bhadra et al. 2020). Investigators have used different waveforms (primarily continuous sinusoidals and discontinuous square waveforms) (Table 1) to achieve KHFAC block (reviewed in Kilgore and Bhadra 2004 (Kilgore and Bhadra 2004)).

**Table 1:** Summary of experimental KHFAC block in the literature. A majority of studies did not state whether amplitudes were peak or peak to peak. The table is arranged in ascending order of frequency range

Authors	Species and nerve	Waveform	Frequency (kHz)	Amplitude range	Amplitude reported as: Peak (p) or Peak to Peak (pp)	Electrode type
Bowman and McNeal 1986	Cat sciatic	Discontinuous square	0.1–10	Not stated	Not stated	Bipolar
Gaunt et al. 2009	Cat pudendal	Continulous Sine	1–10	1–10 mA	Not stated	Bipolar
Kilgore and Bhadra 2004	Frog sciatic	Continuous Sine	1–10	2.4–10 Vpp	pp	Mono- & Bipolar
Tai et al. 2004	Cat pudendal	Continuous Sine Discontinuous Square	1–10	4–10 mA	Not stated	Tripolar
Bhadra et al. 2006	Cat pudendal	Continuous Sine	1–30	1–10 Vpp	pp	Tripolar
Hadaya et al. 2022	Pig paravertebral chain	Not stated	2–20	2–20 mApp	pp	Bipolar
Williamson and Andrews 2005	Rat sciatic	Continuous Sine	3–20	Not stated	Not stated	Bipolar
Cuellar et al. 2013	Rat & Goat L5 nerve root	Continuous Square	3–50	3–15 mA	Not stated	Bipolar
Camilleri et al. 2008	Human vagus	Not stated	5	1–6 mA	Not stated	Not stated
Waataja et al. 2011	Rat vagus	Continuous Square	5	2–7 mA	Not stated	Bipolar
Soin et al 2015	Human sciatic	Continuous Sine	5–10	2–10 V	Not stated	Bipolar
Joseph and Butera 2011	Frog sciatic	Continuous Sine	5–50	1–6 mA	Not stated	Bipolar
Patel and Butera 2015	Rat sciatic	Continuous Sine	5–70	0.2–2 mA	Not stated	Tripolar
Lothet et al 2014	Aplysia pleural-abdominal	Continuous Sine	10	10–15 mApp	pp	Bipolar
Peña et al. 2020	Rat sciatic	Continuous Sine & Square Discontinuous Square	10–20	0.7–3.4 mAp	p	Bipolar
Bhadra and Kilgore 2005	Rat sciatic	Continuous Sine	10–30	2–10 Vpp	pp	Tripolar
Ackermann et al., 2011	Macaque median	Continuous Sine	10–30	2–10 V	Not stated	Bipolar
Roldan et al. 2019	Rat sciatic	Continuous Sine	10–30	Up to 16 Vpp	pp	Bipolar
Bhadra et al. 2020	Rat vagus	Continuous Sine	10–40	2–10 V	Not stated	Bipolar
Pelot and Grill 2020	Rat vagus	Continuous Sine	10–80	0.1–1 mA	Not stated	Bipolar
Ray et al. 2021	Rat sciatic	Continuous Sine	14–26	2–20 Vpp	pp	Bipolar
Eggers et al. 2021	Rat sciatic	Continuous Sine	20	3–5 mApp	pp	Bipolar
Tanner 1962	Frog sciatic	Continuous Shape unknown	20	6.5–14.4 Vp	p	Bipolar
Woo and Campbell 1964	Frog sciatic	Continuous Shape unknown	20	1–5 V	Not stated	Bipolar
Woo and Campbell 1964	Cat sciatic	Continuous Shape unknown	20	1–5 V	Not stated	Bipolar
Fjordbakk et al. 2019	Pig carotid sinus	Not stated	20	12–35 mApp	pp	Bipolar
Ackermann et al. 2010	Rat sciatic	Continuous Sine	40	9–13 mA	Not stated	Bipolar
Patel et al 2017	Rat vagus	Continuous Sine	40	1.5–2 mAp	p	Bipolar
Jiman et al. 2019	Rat renal	Continuous Sine	50	15 V	Not stated	Bipolar
Dewberry et al. 2021	Rat sciatic	Continuous Sine	50	3 Vpp	pp	Bipolar

One recent paper investigated current-controlled and charge-balanced sinusoids and square waveforms in detail (Peña et al. 2020). Waveforms with different asymmetries and duty cycles were studied both in simulations and in-vivo experiments. The nerve block electrodes were fabricated from platinum-iridium. The investigators used sine and square waveforms at the two frequencies of 10 and 20 kHz. They found that sine waves had higher block thresholds than square waveforms but used less power at block threshold. Block thresholds had an inverse relationship with the duty cycle of rectangular waveforms irrespective of waveform asymmetry. Onset responses were not consistently affected by waveform shape, but onset responses were smaller at amplitudes well above block threshold.

KHFAC waveforms are being used clinically in the VBLOC system for weight reduction (Camilleri et al. 2008), and for post-amputation neuroma pain reduction (Neuros Medical, Cleveland, USA) (Soin et al. 2015). However, widespread clinical applications systems have not yet been initiated due to a few remaining issues. The primary characteristic of KHFAC block that limits human use is the initial nerve activity that is generated when the KHFAC is first delivered to a nerve. This activity, termed the “onset response”, has been described by many investigators and has been shown to be much more pronounced in mammals than in amphibians (Kilgore and Bhadra 2004; Bhadra and Kilgore 2005). The onset response has two phases in motor block, Phase I being a summated twitch, and Phase II a period of continued firing. These phases are followed by partial or complete block (Bhadra and Kilgore 2005). The onset response is measured using two parameters; the onset peak and the force-time integral. The first measure focuses on the Phase I response and the second on the Phase II response. In mammals, the onset response can last for many seconds, sometimes even minutes, and produces activity in both motor and sensory nerves (Woo and Campbell 1964; Bowman and McNeal 1986; Kilgore and Bhadra 2004; Bhadra and Kilgore 2005).

For most potential human applications of KHFAC block, the onset response would produce undesirable sensations or movements. Therefore, it is important to identify KHFAC waveform characteristics that reduce or eliminate this activity. Bhadra and Kilgore (2005) (Bhadra and Kilgore 2005) demonstrated that the onset activity can be reduced in a continuous sinusoidal waveform by increasing frequency and amplitude, up to 30 kHz and 10 Vpp, which were the highest values tested. While an amplitude ramped waveform has been suggested for elimination of the onset (Andrews 2004; Tai et al. 2005a), it has been shown through modeling and experiments in mammalian nerve that an amplitude ramp in fact exacerbates the onset response (Miles et al. 2007). In this manuscript, we evaluate whether waveform shape can also influence the size of the onset response.

Another aspect of KHFAC block that has not been fully explored is the physiological response of the nerve to continuous/chronic delivery of the KHFAC waveform. It has been demonstrated that the key predictors of changes in nerve conduction in electrical stimulation are the charge/cycle and the charge/cycle density (McCreery et al. 1990). Based on the effect of electrical stimulation on nerve response, we expect that minimizing the charge delivery to the nerve will minimize adverse tissue responses. Minimizing charge delivery will also reduce the power required for KHFAC block, thus maximizing battery life for implanted block applications. Therefore, it is desirable to evaluate waveform shape to determine if charge delivery can be minimized while still maintaining a consistent nerve conduction block.

Traditionally, KHFAC waveforms have been applied using bare platinum electrodes. Recent advances in direct current nerve block have shown the usability of high capacitance or high surface area electrodes to reduce or eliminate irreversible Faradaic reactions and ensure nerve safety (Vrabec et al. 2016). A recent publication demonstrates the use of a carbon monopolar electrode, using a combined direct current (DC) and KHFAC waveform to minimize the onset response (Vrabec et al. 2013; Eggers et al. 2021). However, one unanswered question is whether the KHFAC onset response using these newer electrodes differs from that produced using bare platinum electrodes.

**Fig. 1:**
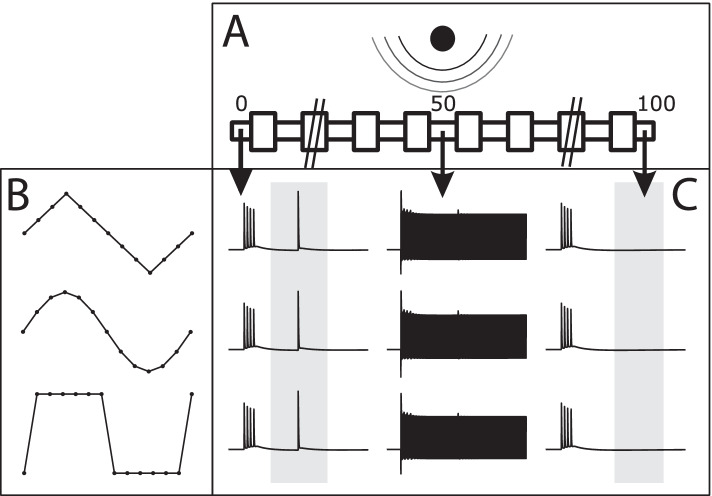
The simulation environment is setup with a point source electrode 1 mm from a 101 node axon (shown in subfigure **A**). The three tested waveforms are shown in subfigure **B** with their 1/12 period discretization. Each waveform was able to achieve block at varying amplitudes, and is shown in voltage plots in subfigure (**C**). The left column of transmembrane voltage plots shows the voltage of node 0 over time with a test pulse sent as seen in the blue indicator box. The center column shows the voltage over time of the central node (node 50). The right column shows the voltage of node 100 over time and the red indicator box showing where no action potentials are seen, indicating that the test pulse was successfully blocked

In the present study, we use computer modeling and in-vivo experiments to specifically compare block thresholds, onset activity and the charge/phase required to produce a complete motor nerve conduction block using continuous charge balanced sinusoidal, triangular and square waves (Fig. 1). We compare two types of bipolar electrodes (bare platinum and carbon coated) and explore the frequency range of 10 kHz to 60 kHz.

## Methods

### Simulation methods

Simulations were carried out using the NEURON simulation environment version 7.6.2 (Hines and Carnevale 1997). Additional code was written using the Python interface to NEURON (Python version 3.6 & 3.7) (Hines et al. 2009), to improve performance. The model was based on a mammalian myelinated axon (McIntyre-Richardson-Grill (MRG) model) originally developed by McIntyre et al. (2002) (McIntyre et al. 2002) and was improved with a frequency-dependent membrane capacitance developed by Howell et al. (2015) (Howell et al. 2015) (Table 2 and Fig. 1).

**Table 2:** Parameters used for MRG simulation of peripheral mammalian axons

Parameters	Values
Node length	1 μm
Myelin attachment paranode length	3 μm
Main paranode length	Diameter dependent
Internodal section length (× 6)	Diameter dependent
DC capacitance (C-DC-)^a^	2 μm cm − 2
Infinte frequency capacitance (C∞)^a^	1.1 μm cm − 2
Myelin capacitance	0.1 μm cm − 2
Axoplasmic resistivity	70 Ω cm
Periaxonal resistivity	70 Ω cm
Myelin conductance	0.001 S cm − 2
Myelin attachment paranode conductance	0.001 S cm − 2
Main segment paranode conductance	0.0001 S cm − 2
Internode segment conductance	0.0001 S cm − 2
Maximum fast Na + conductance	3 S cm − 2
Maximum persistent Na + conductance	0.01 S cm − 2
Maximum slow K+ conductance	0.08 S cm − 2
Nodal leakage conductance	0.007 S cm − 2
Na + Nernst potential	50 mV
K+ Nernst potential	−90 mV
Leakage reversal potential	−90 mV
Resting potential	−80 mV
Temperature	37 °C

^a^Parameters of the frequency-dependent membrane capacitance (Howell et al. 2015)

A 101 node model was used (nodes numbered 0 to 100). The block threshold test was conducted with a stimulating internal electrode at node 0 and recording electrode at node 99. The KHFAC waveforms were injected with a point source electrode 1 mm perpendicular from the center of the axon, which puts it above node 50. Three KHFAC charge-balanced waveforms (sinusoidal, square, and triangular) injected as currents, were tested to measure their respective block thresholds. Block threshold values were investigated for fiber diameters 7.3, 8.7, 10, 11.5, 12.8, 14, 15, and 16 μm. Frequencies of each waveform varied from 4 to 10 kHz in 1 kHz increments, and from 10 to 60 kHz in 5 kHz increments. Frequencies below 10 kHz were simulated with a time step equal to 1/24th the period of the waveform, and frequencies 10 kHz and above were simulated with a time step equal to 1/12th the period of the waveform. All waveforms at all frequencies were tested on all diameters. The determination of the block threshold was performed with multiple simulation runs, where the KHFAC was initiated at t = 10 ms, and a test pulse was generated at t = 50 ms. (Bhadra et al. 2007). The simulation ran until t = 100 ms. The recording electrode monitored the arrival or block of the test pulse and used that information to respectively increase or decrease the amplitude of the KHFAC for the subsequent simulation run. The process was continued as a binary search (Roldan et al. 2019) to determine the minimum amplitude sufficient to block with an 0.5 μA peak to peak resolution (Bhadra et al. 2007; Ackermann et al. 2011a). Block threshold values were then transformed to calculate charge per phase.

A single cathodic half period of all waveforms at all frequencies was also simulated with a point source electrode 1 mm from the axon opposite node 50 to find activation thresholds. Each cathodic half period activation threshold was captured using a binary search to find the minimum amplitude that created an action potential measured at node 2. The same time steps were used for the activation threshold tests as were used in the block threshold tests. An extra set of 10 kHz simulations were run at block threshold for each waveform with the 10 μm diameter axon model. From these simulations, all nodal voltages and gating variables were saved for further analysis to compare between the three waveforms. The values of each node (voltage and gating variables) were averaged across the entire period of the waveform (12 time steps) and then compared across waveforms.

Average current was calculated as BT (mApp) x waveform scalars (1 for square, 0.637 for sinusoids, and 0.5 for triangle). Charge per phase was calculated as average current x half phase width.

### Experimental procedure

#### In-vivo protocol

**Fig. 2:**
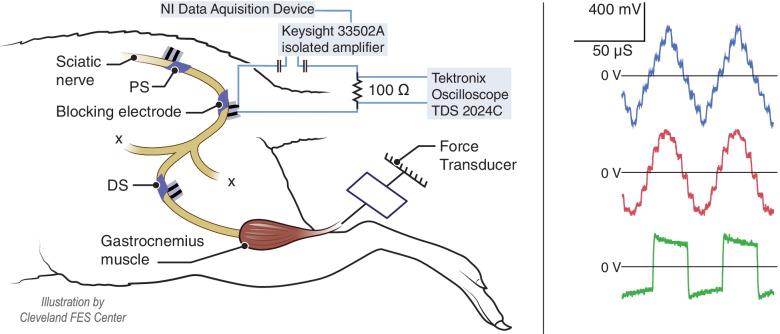
Left: In-vivo experimental setup. A bipolar electrode was placed on the proximal portion of the sciatic nerve to elicit twitches from the gastrocnemius muscle. A second bipolar electrode was placed distally on the nerve. The block electrode was placed between the two stimulating electrodes on the sciatic nerve. The gastrocnemius tendon was attached to a force transducer to record muscle twitches. The blocking waveforms (12 samples per cycle) were delivered from a data acquisition device via an isolated amplifier. The voltage across a 100 Ω series resistor was recorded using an oscilloscope in order to measure peak current at BT. Right: The voltage across a series resistor (100 Ω) in the return path was measured on a high-impedance oscilloscope. Examples are at BT for 20 kHz

Acute experiments were conducted on twelve adult rats (Sprague-Dawley) of weight approximately 400 g. Animals were induced and maintained under anesthesia with isoflurane (1–3%) over the course of the experiment, which typically lasted 7–8 hours. All protocols involving animal use were approved by Case Western Reserve University’s Institutional Animal Care and Use Committee. Briefly, force was recorded from the gastrocnemius-soleus muscle using an in-line force transducer (Entran, Fairfield, NJ) to measure isometric muscle force (resolution .005 N, Bhadra and Kilgore 2005). The left hindlimb was shaved, and an initial incision was made over the sciatic nerve near the iliac crest. The superior gluteal nerve was cut and a proximal stimulation (PS) bipolar bare platinum cuff electrode was placed on the sciatic nerve. A second incision exposed the sciatic nerve near the bifurcation of the tibial and common peroneal nerves. The common peroneal nerve was cut, and a blocking electrode was placed on the sciatic nerve. The separation between the PS and block electrode was approximately 10 mm edge to edge. A distal stimulation electrode (DS) was placed distal to the blocking electrode, with an approximately 3 to 5 mm edge to edge separation. Twitches elicited by this electrode were used to monitor any signs of changes in nerve conduction. Blocking electrodes (used to inject the KHFAC conduction block) were bipolar, with either bare platinum or carbon black-coated contacts. The metal contacts were embedded in a silicone sheet that encircled the nerve with a “J-cuff” design, as show in Fig. 2. Platinum foil was encased between two silicone sheets, and windows of 1 × 3 mm were cut to expose the platinum (Foldes et al. 2011). Carbon black coating was applied as described previously (Eggers et al. 2021; Goh et al. 2022). Total charge capacity of each electrode window was determined in-vitro using cyclic voltammetry prior to in-vivo use (Vrabec et al. 2019). Values for the carbon black-coated electrodes ranged from 16.0–50.5mC.

Voltage controlled blocking waveforms (12 samples per cycle) were generated using a custom LabVIEW (version 20) application, and output via a general purpose data acquisition device (USB-6250, NI). A 5x gain was applied by an amplifier (KEYSIGHT, 33502A). This amplifier was isolated from the Grass stimulator that was used to evoke the motor response. A 3-μF capacitor was placed in series with both the output and return lines to remove any unwanted DC offsets from the waveform. This instrumentation has been used in multiple previous experiments (Roldan et al. 2019; Gerges et al. 2010; Ackermann et al. 2010), and select oscilloscope recordings for all frequencies and waveform shapes were analyzed to confirm charge-balance. Gastrocnemius force output data were sampled at a rate of 1 kHz using a CED 1401 data processor (Cambridge Electronic Design (CED) Ltd., UK) and displayed/recorded on a laptop using Spike2 (version 8) software (CED). The impedance of the blocking electrode was measured at 10 kHz in 9 animals (bare platinum mean 860 ± 172 Ω; carbon black-coated mean 913 ± 232 Ω (Peña et al. 2020). The voltage across a series resistor (100 Ω) in the return path was captured and stored on a high-impedance oscilloscope (Sample rate 100 MHz, Tektronix TDS 2024C, Beaverton, OR, USA). These measurements were taken during the ‘onset at block threshold’ trials (described below).

#### Experimental protocol

**Fig. 3:**
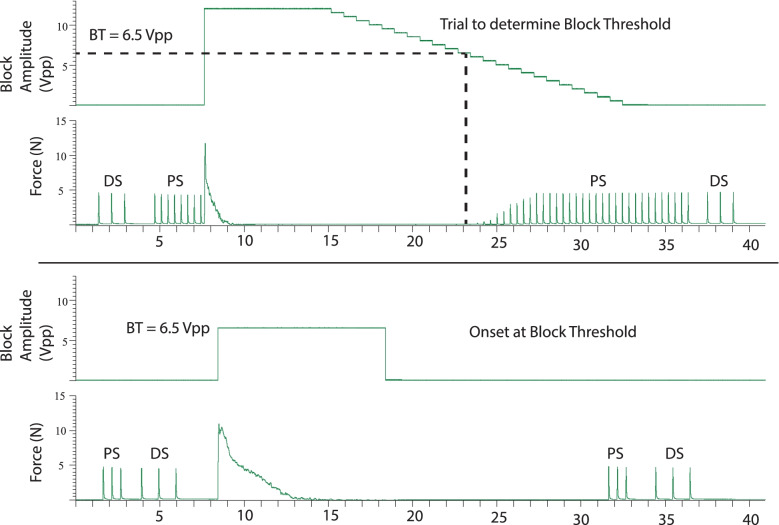
Force output to show paired trial of BT determination and onset at BT. Example of a pair of trials that were performed for each randomized frequency-waveform combination. For each trial, the upper plot is the blocking amplitude, the lower trial is the force output from the gastrocnemius muscle. See Methods section for description

The thresholds for the PS and DS stimulating pulses were mapped at the beginning of the experiment, and remapped between sets, to ensure maximal muscle recruitment. Saturation threshold was the lowest stimulation amplitude that generated the maximal muscle twitch force. Three charge balancedcontinuous waveforms (sine, square and triangular) were tested at 6 frequencies (10, 20, 30, 40, 50 and 60 kHz). These 18 combinations were repeated three times in a randomized statistical block scheme. For each combination two paired trials were performed (see Fig. 3): The first trial was to determine the block threshold (BT) (Roldan et al. 2019). BT is defined as the minimum amplitude that elicits complete block of gastrocnemius twitch force. This was determined by initiating complete block at a supra-threshold KHFAC amplitude level for 10 s, and then stepping down the amplitude with a resolution of 0.5 Vpp every second, until muscle twitches in response to the proximal stimulation reappeared, signifying block failure. The lowest amplitude at which complete block was maintained was determined to be the block threshold. There were approximately 1 minute intervals between successive trials. Each BT trial was followed by a trial to determine the onset responses at the BT. KHFAC was applied for 10 s at BT without any PS during this trial. Occasionally, the onset lasted longer than 10s, so a repeated trial with extended times was performed. In summary, 108 trials were carried out in each animal with 36 trials in each of three independently randomized statistical blocks.

#### Analyses

Analysis of the force output at block threshold was performed in MATLAB (2019a, Mathworks; Natick, MA). The baseline of the force data was zeroed and converted to Newtons based on the calibration of the force transducer. The following measures were determined from the force data: the block threshold, the peak force at block onset, and the force-time integral (i.e., area under the curve of the onset at BT). In addition, the mean heights of the PS and DS force peaks, before and after the application of KHFAC in each trial, were measured. This allowed us to assess any changes in nerve conduction over the course of the experiment. All force measures were normalized to the PS twitch height at the beginning of each trial. Therefore, the primary output measures were block thresholds (in Vpp), normalized peak force at block onset, and normalized force-time integral. The voltage across a serial 100 Ω resistor was used to measure BT currents (mApp) in five of seven bare platinum experiments (three sets each) and four of five carbon experiments (three with three sets, one with one set) (Fig. 2). These data were used to measure charge per half phase at BT and to confirm charge balance of the waveforms.

Statistical modeling and testing were conducted in SAS software version 9.4. A generalized estimating equations (GEE) approach was used, and a series of models to evaluate the association between each of the three primary output measures (BT (Vpp), onset peak height, and onset force-time integral) and two secondary outcomes (BT currents and charge per cycle at BT) and independent variables (electrode type, blocking frequency (kHz) and waveform shape). Models were multivariable in that we evaluated each predictor (electrode type, blocking frequency (kHz) and waveform shape) while controlling for the other two. The modeling approach accounted for the clustering in the data (i.e. random assignment of statistical blocks within animals). We used the Wald test to test the statistical significance of our predictors. The Benjamini–Hochberg method was used as a correction for the False Discovery Rate for multiple hypothesis testing, given five output measures and alpha = 0.05. In secondary analysis, using stratified GEE models we evaluated at each blocking frequency, block thresholds and onset response of the two electrode types, irrespective of waveform shape. We also evaluated at each waveform shape, block thresholds of the two electrode types, irrespective of blocking frequency.

## Results

### Simulation results

**Fig. 4:**
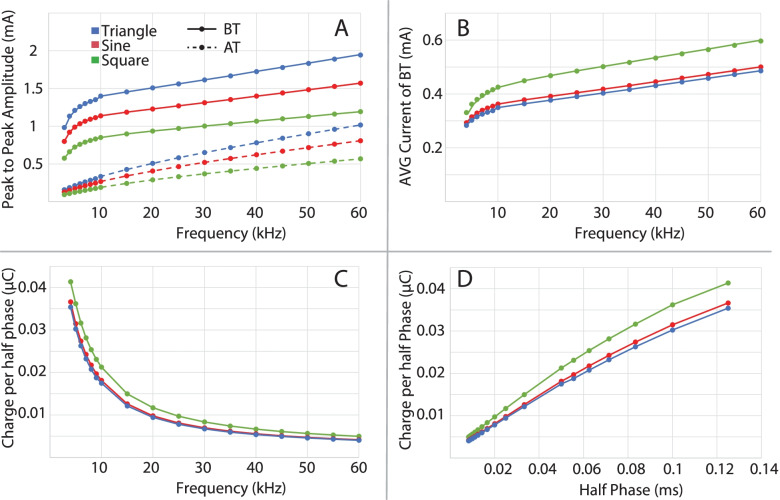
**A** Block Threshold (BT) and Activation Threshold (AT) for a 10 μm diameter axon with triangular (blue), sinusoidal (red), and square (green) waveforms. **B** Average current of all BT values over the frequencies tested. **C** Charge delivered during a half pulse of the blocking waveform. **D** Same values as in C but shown over the half pulse duration (inverse of the frequency scale shown in C)

All three waveforms achieved conduction block at all frequencies and for all axon diameters. Block threshold trends, seen in Fig. 4, of each waveform, mirror those previously seen with a linear trend above 10 kHz (Howell et al. 2015; Bhadra et al. 2007). With all waveform shapes, smaller diameter axons have a higher block threshold at frequencies above 5 kHz, as seen previously (Peña et al. 2020; Howell et al. 2015). The block threshold peak current amplitudes were highest with triangular waveforms, then sinusoidal, and lowest with square waveforms. For average current values (see Fig. 4B) the trends reverse with square waveforms having the highest of the three, followed by sinusoidal, and lastly triangular waveforms. The average current values were then translated to charge per phase, which follows the same order as the average current with square waveforms being the highest, sinusoidal being lower, and triangular waveform the lowest (shown against both frequency and half cycle phase duration in Fig. 4). It is also shown (Fig. 4A) that the activation thresholds from single cathodic half cycles show the same trends as the block thresholds across the three waveforms, with activation thresholds being highest in triangular waveforms, then sinusoidal, and lowest with square waveforms.

**Fig. 5:**
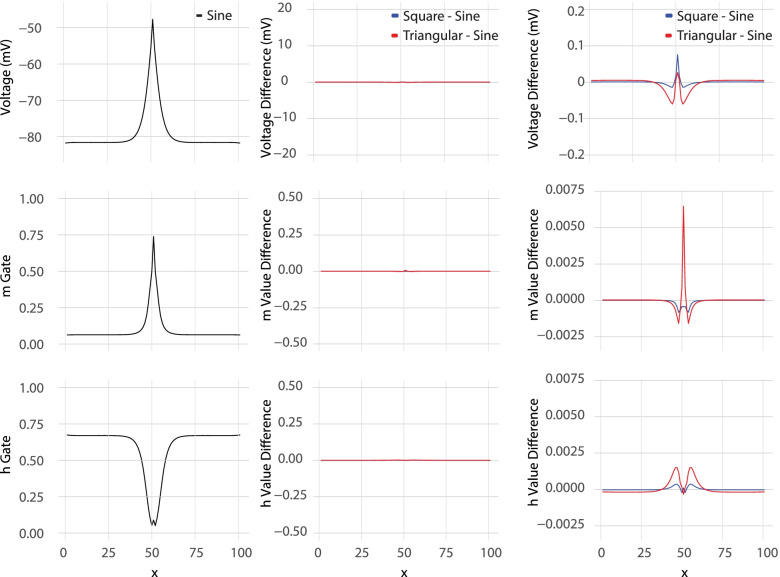
Profile plots showing all transmembrane nodal voltages, m gate, and h gate values in the top, middle, and bottom rows respectively. All values shown are averaged over a single period of the 10 kHz stimulating waveform. The left column shows the profiles from sinusoidal waveform stimulation. The middle column shows the difference between the triangle and sinusoidal stimulation profiles (in blue) and the difference between the square and sinusoidal stimulation profiles (in red). The middle profile differences are shown with the same y-axis scale as the profiles in the left column. The right column is the same data shown in the middle column, but with a 100 times zoom on the y-axis

Looking specifically at the 10 kHz block thresholds of the 10 μm axon with all waveforms, all nodal voltages and gate values were averaged across a full waveform period. All three waveforms create a transmembrane voltage profile, seen in Fig. 5, with the same trends as previously reported (Bhadra et al. 2007). The particular cycle taken for the average is captured at the time a test pulse would have been sent during the block threshold tests. The largest difference between the voltage profiles of any two waveforms was between the sinusoidal and triangular waveforms center node average voltage which was 0.077 mV. This difference is extremely small compared to the over 30 mV of depolarization during KHFAC block and shows that the voltage profile for all three waveforms is nearly identical when averaged over the period. Similarly, the maximum difference between m gate values was 0.0065 between the square and sinusoidal waveforms. The maximum difference between the h gave values was 0.0013 between the square and sinusoidal waveforms at node 45 and 55 (5 from center).

### Experimental results

Complete KHFAC block was achieved at all blocking frequencies, for all waveform shapes, for both electrode types, in all animals (an example is shown in Fig. 3). Seven animals were tested using the *bare platinum* blocking electrode. Three full statistical sets were completed for 6 animals, and 2 sets for 1 animal. Five animals were tested using the *carbon black-coated* blocking electrode. Three full sets were completed for 4 animals, and 1 set in 1 animal. Measurements of the blocking current across a resistor were determined in 5 of the bare platinum experiments, and all 5 of the carbon black experiments.

**Fig. 6:**
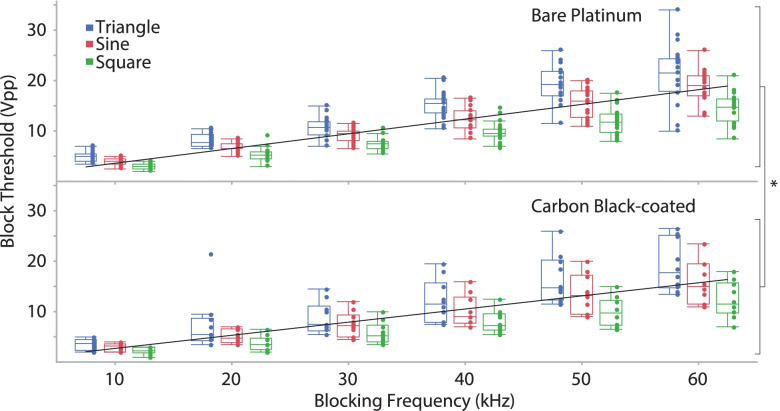
Block thresholds versus frequency for both electrode types. For all waveform shapes, BT increased linearly versus frequency (regression equations for bar platinum and carbon black-coated electrodes, respectively: 0.29*frequency + 0.9, R^2^ = 0.7; 0.26*frequency + 0.01, R^2^ = 0.6). Overall, there is a significant reduction in BT due to application of carbon black coating (* *p* < 0.001)

**Fig. 7:**
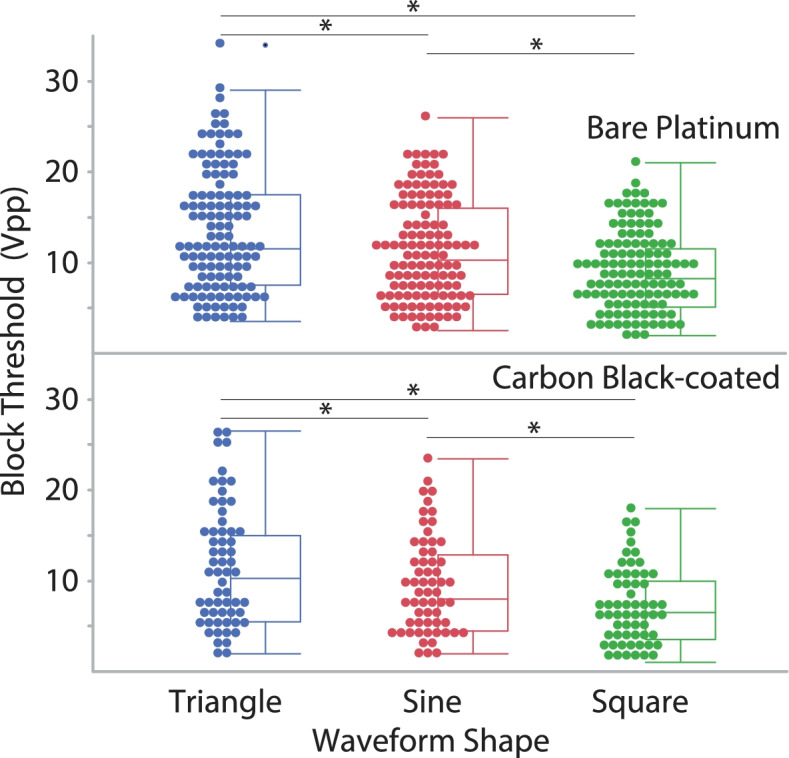
Block Thresholds for each waveform shape. Box plot showing block thresholds for the three waveform shapes. There are significant differences between each waveform shape (* *p* < 0.001)

**Fig. 8:**
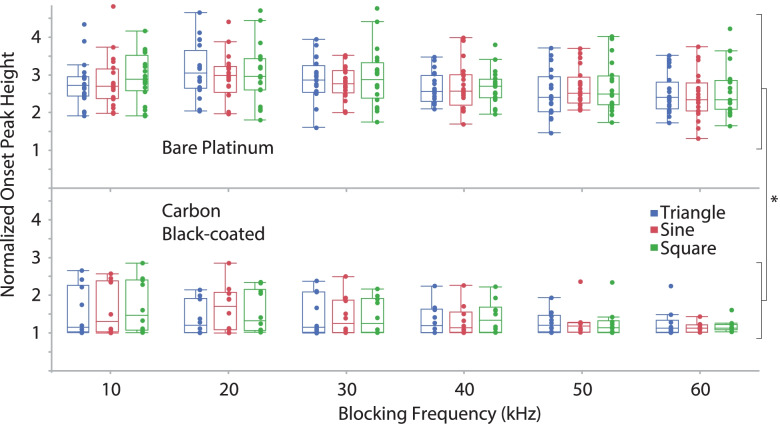
Onset responses for the onset peak for both electrode types. Onset is unaffected by waveform shape. Onset response was significantly affected by blocking frequency (*p* < 0.001) but not waveform shape. Overall (* *p* < 0.001) and at each frequency, carbon black coating yielded significantly lower onset peak height (*p* < 0.001)

**Fig. 9:**
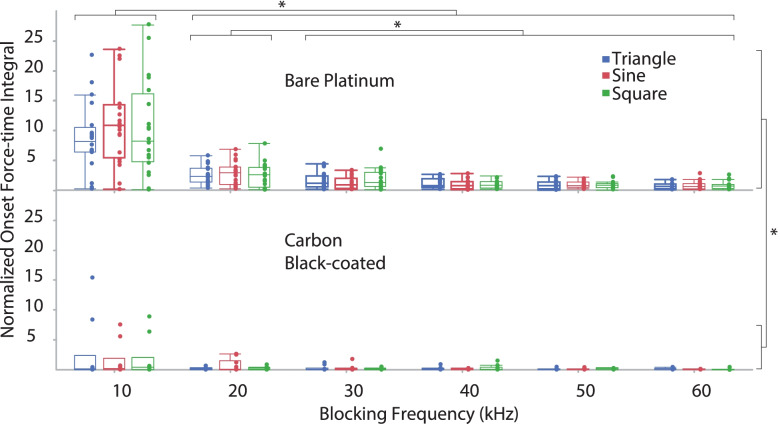
Onset responses for the force-time integral for both electrode types. Onset response was significantly affected by blocking frequency (*p* < 0.001) but not waveform shape. Responses to 10 kHz were significantly larger than all other frequencies (* *p* < 0.001). Responses to 20 kHz were significantly larger than all frequencies greater than 20 kHz (* *p* < 0.001)

**Fig. 10:**
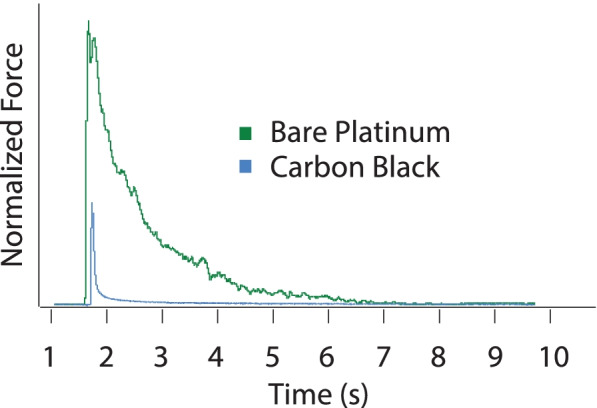
Example of raw force output comparing carbon with bare platinum. Representative examples, based on median force-time integral, of onset at BT. Square blocking waveform at 20 kHz, comparing bare platinum (green) and carbon black-coated platinum (blue). Force normalized to PS peak height, onset time staggered for clarity

For all waveform shapes, BT increased linearly versus *frequency* (regression equations for bare platinum and carbon black-coated electrodes, respectively: 0.29*frequency + 0.9, R^2^ = 0.7, *p* < 0.0001; 0.26*frequency + 0.01, R^2^ = 0.6, *p* < 0.0001) (Fig. 6). There were significant differences in the BT for different *waveform shapes* (*p* < 0.001) (Fig. 7). The triangle waveforms had the highest block thresholds, followed by the sinusoidal and then the square. There was a significant difference between the two *electrode types* (*p* = 0.016), as the BT for carbon black was lower than for the bare platinum. For block thresholds, we found significant differences between *electrode types within each waveform shape*, irrespective of blocking frequency. The BT response was significantly reduced by the application of the carbon black coating within sinusoidal (*p* < 0.001), triangular (*p* = 0.031) and rectangular (*p* < 0.001) waveform shapes. Furthermore, for BT we found a significant difference in *electrode type over frequency* (significantly reduced by the application of the carbon black coating) at 10 kHz through 40 kHz (respectively for 10, 20, 30 and 40 kHz *p* < 0.001, < 0.001, 0.013 and 0.010) Results at 50 kHz (*p* = 0.078) and 60 kHz (*p* = 0.054) were not significant. The onset response (both the onset peak height shown in Fig. 8, and the force-time integral, shown in Fig. 9) was significantly affected by blocking frequency (*p* < 0.001 for both onset peak height and force-time integral). For the force-time integral, 10 kHz responses were significantly larger than all other frequencies (*p* < 0.001, Wald test comparison). Also, 20 kHz responses were significantly larger than all frequencies greater than 20 kHz (*p* < 0.001, Wald test comparison). Onset was unaffected by waveform shape (*p* = 0.106 and *p* = 0.266 for onset peak height and force-time integral, respectively). The onset response was significantly reduced by the application of the carbon black coating (*p* < 0.001 for both onset peak height and force-time integral), a representative example is shown in Fig. 10. At each blocking frequency, the onset response was significantly reduced by the application of the carbon black coating (*p* < 0.001) at every frequency for onset peak height and similarly for force-time integral.

**Fig. 11:**
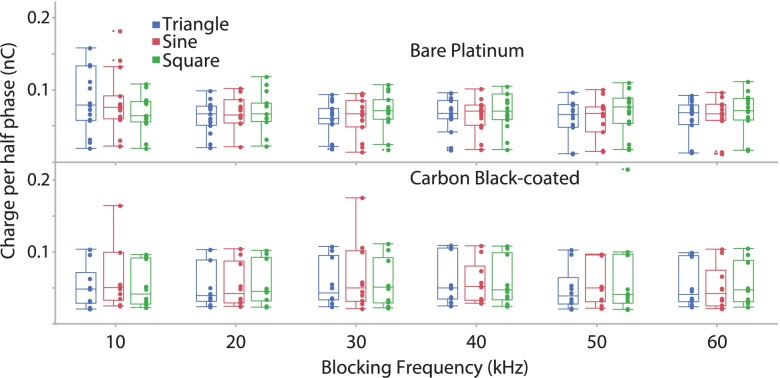
Charge per half cycle at block threshold. There was an overall significant effect of blocking frequency (*P* < 0.0001). This effect was due mainly to the 10 kHz data, and results are suggestive of the exponential trend that was predicted by the MRG model. The application of carbon black coating did not cause a significant reduction in charge required to maintain block

For charge per half phase (Fig. 11), there was an overall significant effect of *blocking frequency* (*P* < 0.0001). This effect was due mainly to the 10 kHz data, since the significance without the 10 kHz data dropped to *P* = 0.0163. Comparisons between the 10 kHz data and those at each other frequency were mostly significant (*P* = 0.0015, 0.0413, 0.055, 0.0048, 0.0002 for 20–60 kHz, respectively). These results are suggestive of the exponential trend, with respect to blocking frequency, that was predicted by the MRG model (Fig. 4C). For *waveform shape*, the Wald test comparison was indicative of a significant effect (*P* = 0.0298). However, there was considerable variance and no constant pattern across blocking frequency in the relationship between waveform shape and charge per half phase. Future studies should evaluate this relationship further. There was no overall significant effect of *carbon block coating* (*P* = 0.1346). The higher variability in the carbon black data is possibly due to variability in Q values because electrodes were coated prior to every experiment, whereas the same bare platinum was used for all experiments and had a stable Q value.

## Discussion

Complete motor nerve conduction block was achieved for the whole range of KHFAC blocking frequencies (10–60 kHz), and for all three continuous waveform shapes (triangular, sinusoidal, and square). In isolated frog sciatic nerve experiments it was previously demonstrated that block could occur at least as low as 2 kHz (Kilgore and Bhadra 2004). However, in the rat sciatic nerve preparation, block below 10 KHz is characterized by a prolonged and intense onset activity (Bhadra and Kilgore 2005). This prolonged activity results in muscle fatigue and adversely affects the experiment. Therefore, we do not routinely test block in these ranges. Most KHFAC publications have tested in the range of 5 kHz to 40 kHz (Table 1). However, block can be obtained experimentally even above 60 KHz (80 kHz tested by Pelot and Grill 2020 (Ackermann et al. 2010)). As shown by both the simulation and experimental results, the threshold for block increases linearly with increasing frequency. However, both simulations and experiments show that the BT to frequency relationship is similar across all three waveform shapes. Furthermore, this was the case for both electrode types: bare platinum and carbon black-coated platinum.

The BT relationship to blocking frequency was linear for all waveform shapes and both electrode types. Previous descriptions of this characteristic have been shown with platinum electrodes (Ackermann et al. 2011b), a narrower frequency range (Bhadra and Kilgore 2005; Ray et al. 2021; Bhadra et al. 2006; Pelot and Grill 2020) and for two waveform shapes (Peña et al. 2020). Here we show that this holds true for all three waveforms (Fig. 5 (simulation) and 6 (experimental)) and two electrode types with different surface properties.

The block threshold was utilized as a key metric to describe the characteristics of KHFAC block (Roldan et al. 2019). Block threshold may be evaluated in various ways, e.g., as peak to peak voltages/currents and average voltages/currents. It is also useful to describe block threshold in units of charge per phase of the waveform (Bhadra et al. 2007). Previous modeling has shown that the charge per cycle at block threshold drops significantly with increasing frequency even though the peak current at BT is higher for higher frequencies (Bhadra et al. 2007). Here, we present updated simulation data showing the same trend, from a model adapted for high frequency stimulation. The in-vivo data from the current study, however, does not match the simulations in thatthere was no significant effect of blocking frequency above 10 kHz. This may be due to the difference in an idealized charge per phase in the simulation as opposed to the measured charge per phase in the experiments. In the simulations, the sharp rise in charge occurs at 10 kHz and lower. However, as stated in the introduction, the in-vivo frequency range could not practically extend below 10 kHz.

The effect of frequency on charge per phase at BT has important implications for the study of the safety of KHFAC block, since the charge per phase per electrode area has been shown to be an important parameter for the safety of conventional square wave stimulation pulses (McCreery et al. 1990). To date, only a few literature reports on experimental KHFAC block have included this type of measurement (Peña et al. 2020), although block thresholds have been documented in modeling studies (Williamson and Andrews 2005; Tai et al. 2005a; Tai et al. 2005b). The relationship between block thresholds and KHFAC phase width (half-cycle pulse width) is similar to the well-established strength-duration relationship for nerve activation (Bhadra et al. 2007). The simulation results presented in this study (Fig. 4) also show that the relationship between charge per phase at block threshold and half-cycle pulse width is nearly linear and shows similarities to charge-duration plots for nerve activation (Boinagrov et al. 2010). The similarity between the threshold-phase width relationship for block, and the threshold-duration relationship for activation is hypothesized to be the result of a common mechanism underlying nerve activation and KHFAC blocking, specifically membrane depolarization (Bhadra et al. 2007).

Our simulation results also show that waveform shape influences the charge per phase required for block. In all cases, charge delivery is minimized with the triangular waveform (Fig. 4). Our experimental results show the same trend (Fig. 11). Minimizing the charge per phase is likely to be an important factor determining the safety of KHFAC waveforms for clinical nerve block applications (McCreery et al. 1990).

Simulations also showed the same voltage profile for all waveform shapes and matched that previously seen in Bhadra et al. (2007) (Bhadra et al. 2007), even with the inclusion of the frequency-dependent membrane capacitance. While sinusoidal waves only have a single frequency component, the triangle and square waves have an infinite series of higher harmonics, making the inclusion of a frequency-dependent membrane capacitance particularly significant. The relatively small difference between the voltage and state profiles across waveforms shows that there is a similar effect on the axon, leading to the constant activation of the sodium gate in a quasi-steady state as a common mechanism of block across waveforms (Eggers et al. 2021; Ackermann et al. 2011a).

One limitation is that the simulations were conducted with a monopolar point electrode while experiments were performed with bipolar electrodes. We recognize that bipolar simulations with non-point-source electrodes, using Finite Electrode Modeling would have been more realistic. However, we did not intend to find block thresholds that directly matched the experimental results. Our aim was to inspect the behavior of KHFAC using a simple electrode configuration. With this we found similar trends between simulations and experiments, including the block threshold differences and relationships to frequency. The model also revealed behaviors of the h and m gates which we believe would be similar with bipolar simulations since the KHFAC block is produced independently at each electrode contact.

The paired threshold plus onset trial design provides a consistent comparison of onset magnitude. Specifically, the trial to identify the block threshold at any particular electrode/frequency/waveform combination starts at an amplitude that is higher than the block threshold. Therefore, the onset produced in that trial underestimates the extent of the onset. To be consistent we used a second trial at the block threshold amplitude identified in the preceding trial. This ensured that the onset response measurement was always performed at the block threshold of that particular electrode/frequency/waveform combination.

The onset response was quantified in two ways: onset peak height and force-time integral. We have previously demonstrated that the KHFAC onset in motor block has two phases: Phase I being a summated twitch, and Phase II a period of continued firing (Bhadra and Kilgore 2005). The second phase in the onset phenomenon is the period of tetany or repetitive fasciculation of parts of the muscle (Bhadra and Kilgore 2005) caused by repetitive firing of the nerve. This activity, measured by the force-time integral, showed a typical relationship in the KHFAC frequency-amplitude space, being decreased with combined higher frequencies and higher amplitudes (Bhadra and Kilgore 2005). For both metrics, onset response was unaffected by waveform shape. This is consistent with the results of Peña et al., 2020, who compared square and sinusoid wave onset response using the force-time integral (Peña et al. 2020).

Carbon black has high charge capacitance properties and has been used to safely deliver DC to block nerves (Vrabec et al. 2016; Vrabec et al. 2019), and as part of a combined DC/KHFAC waveform to block KHFAC onset response (Eggers et al. 2021). To date, there has not been a direct comparison of the KHFAC properties of carbon-black coating with bare metal electrodes. In the present study it was demonstrated that the application of a carbon black coating to platinum nerve cuff electrodes considerably reducing onset peaks and onset force-time integrals (Figs. 6, 8, 9 and 10). Five different metal and metal alloy electrodes were compared in a previous KHFAC study (Patel et al. 2018) and found no significant differences in BT or onset between the materials. In our present study, the carbon black coating increases the capacitance of the electrode for the same size electrode contact. Since the charge per phase data showed no significant reduction in BT for carbon black. It is possible that the charge density properties of carbon black-coated electrodes are responsible for the improved onset responses, as well as the reduced BT (Vpp).

## Conclusion

KHFAC consistently blocks over the entire range of 10–60 kHz. We show that increasing frequency leads to higher BTs and lower onsets. We show that waveform shape affects the BTs (square, sinusoidal, triangular in increasing BTs) but not the onset responses. We show that carbon black coating significantly reduces both the BT and the onset response. The charge per phase is affected by frequency and less so by waveform shape but not by electrode material.

This research suggests that future investigation of carbon black or other high charge capacity electrodes may be useful in achieving block with lower BTs and onsets. Efficient delivery of these waveforms to achieve nerve block clinically would favor waveforms with lower charge requirements leading to lower power demands. The other key determination will be the identification of the most promising frequency range for KHFAC. There will be a similar tradeoff here since lower frequencies offer lower thresholds but higher charge per phase. We conclude that both sinusoidal and square waveforms at frequencies of 20 kHz or higher would be optimal.

## Data Availability

The datasets used and/or analyzed during the current study are available from the corresponding author on reasonable request.

## Abbreviations

KHFAC: Kilohertz frequency alternating current
DC: Direct current
MRG: McIntyre-Richardson-Grill model
Vp: Volts peak
Vpp: Volts peak to peak
mApp: Milliamperes peak to peak
DS: Distal stimulation
PS: Proximal stimulation
BT: Block threshold
GEE: Generalized estimating equations
